# Ganjam virus/Nairobi sheep disease virus induces a pro-inflammatory response in infected sheep

**DOI:** 10.1186/1297-9716-43-71

**Published:** 2012-10-19

**Authors:** Abid bin Tarif, Lidia Lasecka, Barbara Holzer, Michael D Baron

**Affiliations:** 1 The Pirbright Institute, Ash Road, Pirbright, Woking, Surrey, GU24 0NF, United Kingdom

## Abstract

Partly due to climate change, and partly due to changes of human habitat occupation, the impact of tick-borne viruses is increasing. Nairobi sheep disease virus (NSDV) and Ganjam virus (GV) are two names for the same virus, which causes disease in sheep and goats and is currently known to be circulating in India and East Africa. The virus is transmitted by ixodid ticks and causes a severe hemorrhagic disease. We have developed a real-time PCR assay for the virus genome and validated it in a pilot study of the pathogenicity induced by two different isolates of NSDV/GV. One isolate was highly adapted to tissue culture, grew in most cell lines tested, and was essentially apathogenic in sheep. The second isolate appeared to be poorly adapted to cell culture and retained pathogenicity in sheep. The real-time PCR assay for virus easily detected 4 copies or less of the viral genome, and allowed a quantitative measure of the virus in whole blood. Measurement of the changes in cytokine mRNAs showed similar changes to those observed in humans infected by the closely related virus Crimean Congo hemorrhagic fever virus.

## Introduction

Nairobi sheep disease (NSD) was first identified at the beginning of the 20^th^ century by Montgomery as a disease affecting sheep and goats in parts of Kenya [1]. It has since been identified in several places in East Africa. A similar disease has also been reported in north east India, where it was called Ganjam [2]. The recent application of molecular sequencing techniques to the viruses that cause these diseases (NSDV and GV, respectively) revealed that they are the same virus [3,4], with different strains existing on the two continents. Whether the virus has existed for an historically long time in both places, or is a relatively recent import from one part of the world to another has yet to be determined. It is possible that the virus was imported to Africa from India as a consequence of the same kind of livestock movement that introduced rinderpest virus to Africa in the 1880 s [5].

The virus is spread by hard (Ixodid) ticks, and appears to be dependent on the tick vector for dissemination, with no direct transmission between animals. This obligate vector step may explain why the virus is not seen as a major economic threat, since young animals in endemic areas tend to be protected by maternal antibodies through the period where they are first exposed to the virus via a bite from an infected tick, after which they have their own immune protection. The disease tends to be only noticed on introduction of naive livestock into an endemic area, e.g. for the purposes of improving local breeds by crossing. The disease that ensues is regarded as one of the most pathogenic in small ruminants, with mortality rates as high as 90%; animals die from acute haemorrhagic fever [1,6]. Disease is only seen in sheep and goats, with no disease seen or viraemia detected when cattle, buffalo, equids or other mammals are infected [1,7], although the limitations of early virus detection methods (pathogenesis in neonatal mouse brains) have to be borne in mind. NSDV was originally seen as a disease with a relatively restricted distribution, a distribution largely dependent on that of the *Rhipicephalus appendiculatus* tick [1,8]; in contrast, GV has been reported predominantly in *Haemaphysalis* species in India [7,9]. Recent studies, especially using molecular detection techniques, have found the virus in tick samples from a much wider geographical area, and it now appears that it is distributed over most of the Indian sub-continent as well as much wider in East Africa than the restricted area in Kenya originally reported [9].

NSDV/GV is a bunyavirus of the genus *Nairovirus*; other members of the genus include *Dugbe virus* and *Kupe virus*, both isolated from cattle ticks in East Africa, and the human pathogen *Crimean Congo hemorrhagic fever virus* (CCHFV). CCHFV is another tick-borne virus which appears to be spreading, with increasing outbreaks in Russia, Turkey, India and Pakistan and recent detection of the virus in tick samples from Spain [10]. The spread of CCHFV, or at least outbreaks of disease, seems to be a consequence of a combination of changes in land use and climate, leading to increased contact between people and ticks, and possibly changes in the range of the tick vectors as well as their competence to propagate the virus [11]. The range of NSDV/GV may likewise be spreading, and its impact will also increase as we push more and more for breed improvement and maximising land use to manage the increasing global demands for food. For this reason, and because it has promise as a good model system to study the nairoviruses (while work on CCHFV is restricted to BSL4 laboratories, and lacks an in vivo system to study disease), we have initiated work on NSDV/GV with a view to characterising the virus and its pathology.

Early studies described the clinical signs of the disease in detail, as well as establishing the dependence on the tick vector. We have recently shown that the virus can block the actions of both type 1 (interferon α/β) and type 2 (interferon γ) interferons, as well as inhibit the induction of interferon β in infected cells [12]. We report here the results of an initial study of the replication of the virus in sheep and the major cytokine responses in infected animals. We found a fundamentally pro-inflammatory response, with specific differences between responses to a pathogenic and a non-pathogenic virus. As part of the project, we have developed a sensitive, NSDV/GV-specific, real-time PCR assay for detecting viral RNA which may be useful in other labs for screening diagnostic samples where nairovirus infection is suspected.

## Materials and methods

### Viruses and cells

Except where indicated, media and cells were obtained from the Central Sterilisation Unit, this institute. MDBK (Madin-Darby bovine kidney) cells and Vero-SLAM (African green monkey kidney, expressing human SLAM) cells (the gift of Dr Rick De Swart, Department of Virology, Erasmus MC, The Netherlands) were grown in Dulbecco’s modified Eagle’s medium (DMEM) supplemented with 5% foetal calf serum (FCS). Although SLAM was not required for growth of NSDV, Vero-SLAM cells were the Vero cells in general use in our laboratory and it was known that the virus can infect these cells. BHK21/clone 13 (baby hamster kidney) cells were obtained from ATCC (LGC Standards, Teddington, UK) and cultured in Glasgow modified Eagle's Medium (GMEM) containing 10% FCS. PO (sheep, kidney) cells (from the Collection of Cell Lines in Veterinary Medicine (CCLV), Friedrich Loeffler Institute, Riems, Germany) and BSR-T7 (a BHK-derived cell line constitutively expressing T7 RNA polymerase) cells (a gift from Prof K. K. Conzelman) were grown in DMEM medium enriched with 10% FCS. SSF (primary sheep skin fibroblast) cells and BSF (primary bovine skin fibroblast) cells were prepared previously as described by Childerstone et al. [13]. These cells were maintained in Iscove’s modified Dulbecco’s medium (IMDM) (Life Technologies, Paisley, UK) containing 10% FCS. BFA (bovine foetal aortic endothelium) cells were obtained from the European Cell Culture Collection) and grown on Nutrient Mixture F-12 Ham medium (Sigma, Dorset, UK) containing 20% FCS. Primary ovine endothelial cells were either obtained from Dr H-H Takamatsu (The Pirbright Institute) and maintained in IMDM containing 10% FCS or prepared from ovine pulmonary artery and aorta essentially as described [14] and maintained in medium M131 supplemented with microvascular growth supplement (MVGS) (Life Technologies).

The Nairobi sheep disease virus (NSDV) isolate (ND66-PC9) was obtained from Dr Piet van Rijn, Central Veterinary Institute of Wageningen, Netherlands. The Ganjam virus (GV) isolate (IG619, TVPII 236) was obtained from World Reference Center for Emerging Viruses and Arboviruses at the Galveston National Laboratory, and was the kind gift of Prof Robert B Tesh, University of Texas Medical Branch, Galveston, Texas, USA. Virus stocks were grown in BHK21/clone 13 cells using GMEM containing 2% FCS, penicillin (100 U/mL), streptomycin sulphate (100 μg/mL), 2 mM L-glutamine and 5% tryptose phosphate broth solution. The virus titre was determined as the 50% tissue culture infectious dose (TCID_50_) in BHK21 cells. Both strains grew to similar final titres (~10^6^/mL) and were used after two additional passages in BHK cells. Multiplicity of infection (MOI) was calculated as TCID_50_ per plated cell.

### Multi-step growth curves of virus

Cells were plated in 6-well dishes 6-9 h before use, apart from primary endothelial cells, which were plated 18 h before infection to ensure good attachment. Cells were infected with NSDV or GV at a MOI of 0.01; after 1 h incubation at 37°C, 5% CO2, the inoculum was removed, the cells were washed once with growth medium and incubated in fresh medium at 37°C, 5% CO2. At 0, 12, 24, 36, 48 and 72 hours post infection (hpi) samples were frozen at -80°C. Each virus time course was carried out at least in duplicate. When all samples had been collected, they were thawed and centrifuged at 2500 rpm, 4°C for 10 min to remove cell debris. The supernatants were stored at -80°C. The amount of viruses in each sample was determined by titration on BSR-T7 cells (for NSDV) or BHK21 (for GV). CPE (cytopathic effect) was scored at 3-5 days post infection (dpi) and virus titre was calculated as TCID_50_/mL by the Spearman-Kärber method [15].

### Animal study

The animal study described in this paper was subject to full ethical review and licensing under the Animals (Scientific Procedures) Act 1986 of the United Kingdom, and was approved by the competent authority with Project Licence number 70/7014. Six outbred sheep (female Dorset breed animals at 7-8 months of age) were obtained from commercial suppliers. Three animals were infected subcutaneously with 10^4^ TCID_50_ units of either the NSDV or GV isolate at the first passage in BHK 21/clone 13 cells from receipt of samples. The rectal temperature of the animals was measured before the experiment began and each day during the experiment. Blood samples were taken prior to infection and on the indicated days post infection into vacutainers for serum (coagulated blood) and leucocytes (EDTA as anti-coagulant) as well as into Tempus® vacutainers (Life Technologies) for stabilisation of total RNA. Serum samples were separated and stored at -20°C. White cell counts were determined from duplicate samples on the day of sampling, using a Cellometer Auto T4 (Nexcelcom, Lawrence, MA, USA). Red cells were pelleted by centrifugation and the supernatant (essentially plasma plus buffy coat cells) stored at -80°C until used for virus isolation or RNA extraction.

### RT-qPCR of viral RNA and ovine cytokines

RNA was prepared from the whole blood samples in Tempus tubes using the Tempus® Spin RNA Isolation kit (Life Technologies). RNA was extracted from white cell samples using RNeasy mini kits (Qiagen, West Sussex, UK). All oligonucleotide primers were from Sigma. Reverse transcription was carried out as instructed by the manufacturer using Superscript II (Life Technologies) with either genome-specific primer (0.1 pmol/μL final concentration) or Oligo(dT)-Anch ((T)_16_VN) (5 pmol/μL final concentration). cDNA was diluted 4-fold (3-fold if RNA concentration was low) in water and heated at 75°C for 15 min before use in PCR. PCR was performed in 10 μL (initial gradient PCRs) or 20 μL (real-time PCR) reactions using Applied Biosystems SYBR® Green PCR Master Mix (Life Technologies). Real-time PCR reactions were carried out on a Rotorgene 2000 (Qiagen) and analysed using Rotor-Gene software v6; the threshold for determining the Ct was set at normalised fluorescence = 0.01. The PCR program used consisted of a 10 min activation step at 95°C followed by 40 cycles of 15 s at 95°C, 30 s at the appropriate annealing temperature (Table 1) and 30 s at 72°C. Final primer concentrations for each real-time PCR assay were as listed in Table 1. Each reaction contained 15 ng (whole blood) or 3 ng (white cells) of RNA as cDNA.

**Table 1:** PCR primer pairs and reaction conditions used in the work described in this paper

**Target**	**Primer sequences**	**Ta**^**1**^	**[Primer]**^**2**^	**Reference**
NSDV/GV (F1/R1A)	TGACCATGCAGAACCAGATYG	62	300nM	this paper
	GAAACAAGCCTCATGCTAACCT			
NSDV/GV (F2/R2)	GGAGAATGGCAAAGAGGTTGT	64	300nM	this paper
	GTAAATCCGATTGGCAGTGAAG			
NSDV/GV F3b (RT primer)	GTCTTTGAACTYTGACCA	n/a	n/a	this paper
IL-1β	CCTTGGGTATCAGGGACAA	60	300nM	[16]
	TGCGTATGGCTTTCTTTAGG			
IL-4	ACCTGTTCTGTGAATGAAGCCAA	60	300nM	[17]
	CCCTCATAATAGTCTTTAGCCTTTCC			
IL-6	TCCAGAACGAGTTTGAGG	60	400nM	[16]
	CATCCGAATAGCTCTCAG			
IL-8	ATGAGTACAGAACTTCGA	57	300nM	[16]
	TCATGGATCTTGCTTCTC			
IL-10	TGCTGTTGACCCAGTCTCTG	60	200nM	this paper
	AGGGCAGAAAACGATGACAG			
IL-12A	TGGGCATTGTCTGTCTTCTG	60	200nM	this paper
	TTCTTCCAGGGAGGGTTTCT			
IL-12B	GCTGGGAGTACCCTGACACG	61	500nM	[17]
	GTGACTTTGGCTGAGGTTTGGTC			
IL-18	ACTGTTCAGATAATGCACCCCAG	60	300nM	[17]
	TTCTTACACTGCACAGAGATGGTTAC			
Interferon β	CCAGATGGTTCTCCTGCTGTGT	63	300nM	this paper
	GACCAATACGGCATCTTCCTTC			
TNFα	GAATACCTGGACTATGCCGA	60	200nM	[16]
	CCTCACTTCCCTACATCCCT			
TGFβ	GTGGACATCAACGGGTTCAG	60	300nM	this paper
	TGTCCAGGCTCCAGATGTAG			
Interferon γ	CTCCGGCCTAACTCTCTCCT	60	300nM	this paper
	AGGCCCACCCTTAGCTACAT			
GAPDH	GGTGATGCTGGTGCTGAGTA	60	300nM	[16]
	TCATAAGTCCCTCCACGATG			
SDHA	ACCTGATGCTTTGTGCTCTGC	60	200nM	[16]
	CCTGGACGGGCTTGGAGTAA			
G6PDH	CGAGGCTGTGTACACCAAGA	60	300nM	this paper
	ATGTGGTGGAGCAGTGGAGT			

1: Ta: annealing temperature.

2: [Primer]: primer concentration.

### Statistical analysis

Real-time PCR data from the animal experiment was analysed using the General Linear Model form of ANOVA as implemented in Minitab 16 with a model in which the virus used and the days post infection were fixed factors. Due to the loss of some animals at day 7, analysis was restricted to the data from days 0, 2, 4 and 7. The two virus isolates were compared using the ANOVA of the linear model, and the significance of any increase or decrease of transcription on day 2, 4 and 7, compared to the value at day zero, was determined using Dunnett’s correction for multiple comparisons.

## Results

### Characteristics of virus isolates in cell culture

**Figure 1:**
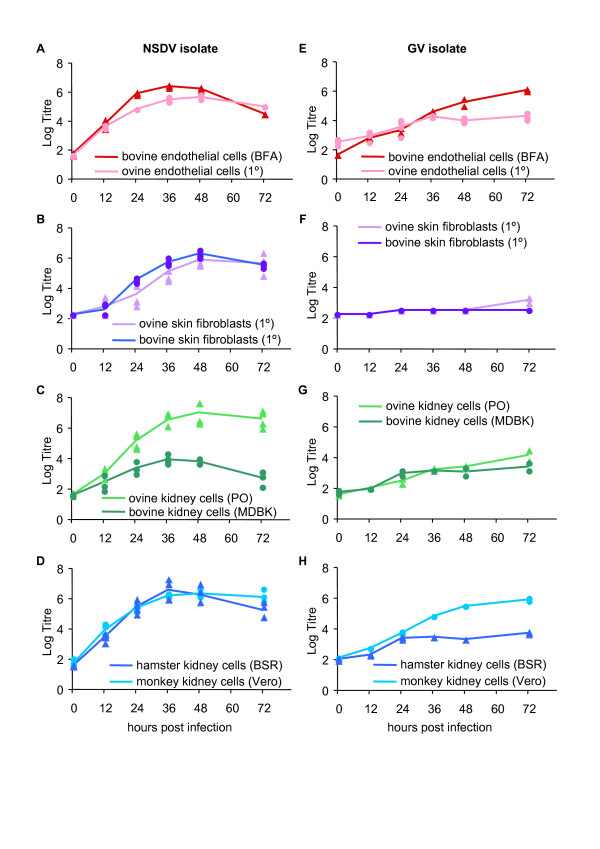
**Growth of NSDV/GV isolates in cultured cells.** The NSDV isolate (**A**-**D**) or the GV isolate (**E**-**H**) were used to infect different primary (1º) or permanent cell lines as described in “Materials and methods”. At the indicated times post infection the cells were frozen and the titre (TCID_50_/mL) of virus in the cell-free supernatant determined. Each experiment was carried out 2-4 times; symbols representing individual experiments may overlap.

Two isolates of NSDV/GV were available to us, one of NSDV Entebbe strain (ND66-PC9) originally prepared by Terpstra from samples taken in Uganda in 1956 [18] and passed 75 times in tissue culture, the other of GV (IG619), originally isolated in India, but with no recorded passage history. Both isolates were found to grow to good titres (10^6^ TCID_50_/mL) on BHK21/clone13 cells, as previously reported for NSDV [6] (data not shown). We assessed their ability to grow in a variety of other cultured cells, both continuous lines and primary cells (Figure 1). The NSDV isolate grew well, to titres of 10^6^ TCID_50_/mL, in all the cell lines tested with the exception of MDBK cells, an established bovine kidney line, where the peak titre was only 10^4^ TCID_50_/mL. This was not due to a species-specific restriction, since the virus grew equally well in bovine and ovine skin fibroblasts, and in bovine and ovine endothelial cells (Figure 1). This isolate grew well also in another hamster kidney-derived cell line (BSR-T7). Cytopathic effect (CPE) was observed in most of the cell types, though it appeared more slowly in the primary skin fibroblasts. In contrast, the GV isolate grew well only in BHK21/clone13 cells, Vero cells or the bovine foetal endothelial cell line, and showed strong CPE only in the BHK cells, which were therefore used for titration of GV stocks. This virus grew poorly in ovine or bovine kidney cell lines, or in primary goat or sheep endothelial cells, and essentially did not grow in the primary ovine or bovine skin fibroblasts (Figure 1). In general NSDV growth peaked at earlier time points (at 36 and 48 h post infection), whereas the GV displayed a slower growth rate, and the virus titre did not peak by the end of the time course. These data suggested that the extended passage of the NSDV isolate in BHK cells has adapted it to cell culture in general; other studies in our laboratory have shown this isolate replicates well in human cells as well (A549 cells). The GV isolate appeared to be significantly more restricted in the cell lines it will enter and replicate in. However, there seems to be no species specific restriction since each virus isolate grew equally well in hamster, monkey and human-derived cell lines as well as in bovine and ovine cells. An interesting observation was that this isolate grew significantly worse in BSR-T7 cells (max titre 10^4^) compared to BHK21/clone13 cells, despite the fact that they are both subclones of BHK cells, and it grew better in Veros than in BSR-T7 cells, despite the observation that both cells are defective in production of type 1 interferon [19,20].

### Development of real-time assay for NSDV/GV genome

**Figure 2:**
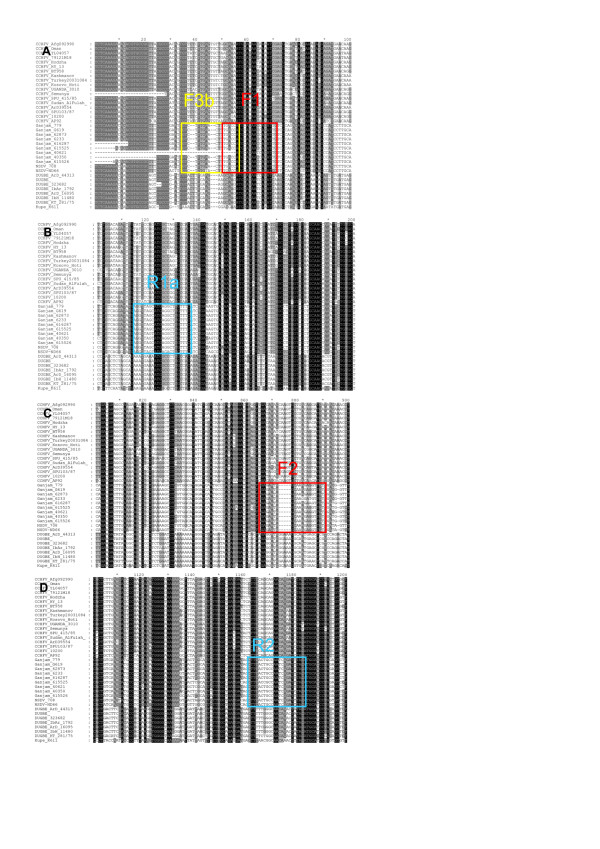
**Identification of specific primers for NSDV/GV PCR.** An alignment of all available S segments for nairoviruses (CCHFV, NSDV, GV, Dugbe virus and Kupe virus) was made and extracted blocks from this alignment are shown to illustrate the differences between NSDV/GV and other nairoviruses at the points selected for use as RT and PCR primers.

**Figure 3:**
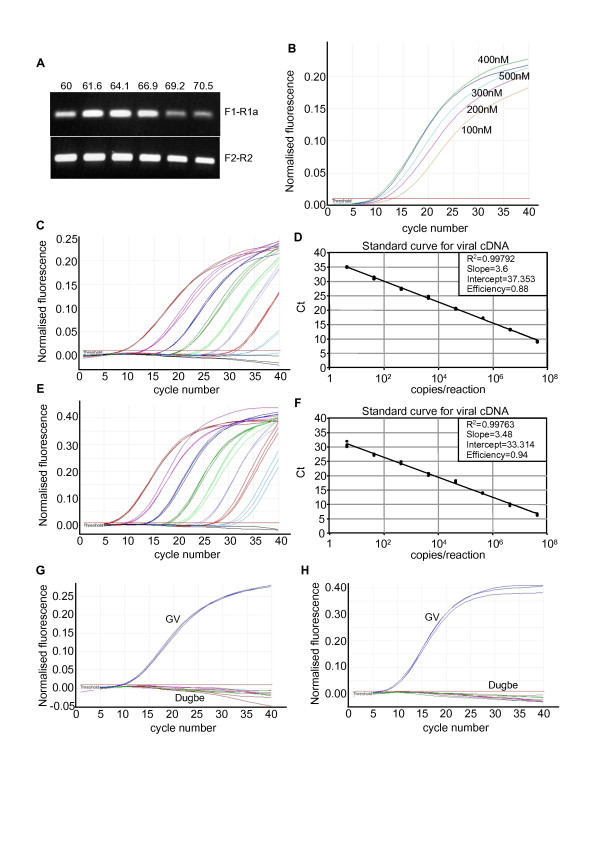
**Optimisation and validation of PCR primers for NSDV/GV detection and quantitation.A**) Example gradient PCRs of F1/R1a and F2/R2 primer pairs. **B**) Example real-time PCR with 100-500nM primer concentration for F1/R1a primer pair. **C**) Sensitivity determination for real-time PCR with F1-R1a primer pair; serial dilutions were made of GV S segment DNA template from 4 to 4 × 10^7^ copies per reaction and the real-time PCR carried out with F1/R1a. **D**) Plot of data from (C). **E**), **F**) Similar sensitivity determination and standard curve for primer pair F2/R2. **G**) Real-time PCR results for F1/R1a primer pair with 4 × 10^7^ copies of GV (blue) or Dugbe virus (brown) S segment; purple and green lines are negative controls. **H**) As (G), but with F2/R2 primer pair.

In order to be able to track and quantitate the growth and spread of the virus isolates in samples taken during animal studies, we developed a real-time PCR based assay for viral RNA. We selected primers based on the available sequences of the S segments of NSDV, GV, CCHFV, Dugbe virus and Kupe virus. The S segment was chosen because there is more extensive sequence data for that segment than the M or L segments, and primers were selected for the reverse transcription (RT) step and for a Sybr Green-based real-time PCR. We used a genome-specific primer (F3b) for the reverse transcription (RT) step as the assay was to be used for quantitation; random hexanucleotide primers, while possibly more sensitive, are not compatible with RNA quantitation [21]. Preliminary tests showed that lower background and higher sensitivity was achieved using a single primer external to the PCR target than by using the same primers for the RT and PCR steps. We sought to find primer sets that were conserved in NSDV/GV but not in any of the other nairoviruses, so that the assay could also be used as a diagnostic for NSDV/GV should the need arise in the future. The location of the primers in the overall alignment are shown in Figure 2, and the sequence of the primers used are listed in Table 1, along with the reaction conditions (annealing temperature and primer concentration). All primer pairs were optimised for annealing temperature by gradient PCR, and the optimal primer concentration determined (e.g. Figure 3B). Primer pairs F1-R1a and F2-R2 both worked well with similar sensitivity (Figure 3C-F), detecting fewer than 10 copies of target. We tested whether the primers would react with Dugbe virus, since this virus is also found associated with livestock. No reaction was seen with a clone of Dugbe virus S segment (Figure 3G-H). No reaction product was seen in samples without template (NTC controls), and this assay was used to measure viral genome RNA in the subsequent animal experiment.

### Real-time PCR measurement of cytokine mRNA levels

A set of primers specific for a range of ovine cytokines was prepared, either using published primer pairs or designed from ovine mRNA sequences taken from the data base. The reaction conditions for each primer pair were optimised as described for the viral RNA assay, using an anchored oligo(dT) oligonucleotide ((T)_16_VN) to prime the RT reactions. Some published primer pairs for specific ovine cytokines were found to have low reaction efficiency, and new primers were designed for those assays. A complete listing of the primers used and reaction conditions for the relevant assays is given in Table 1.

### Pathogenicity and virus growth in animals

**Figure 4:**
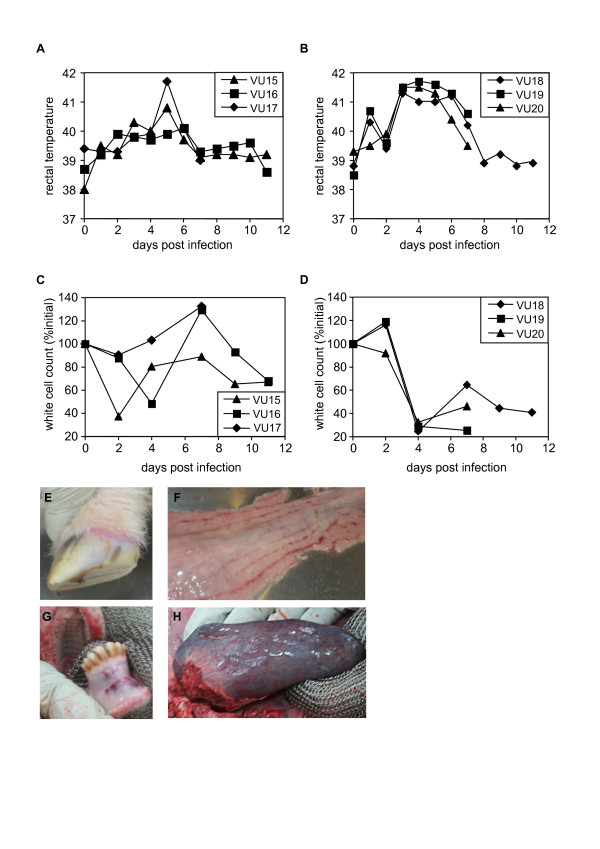
**Effects of NSDV and GV isolates on sheep.** Three animals (VU15-17) were infected with the NSDV isolate and three (VU18-20) were infected with the GV isolate. Animals VU17, VU19 and VU20 were sacrificed at 7 days post infection, when VU19 showed extreme clinical signs. **A**, **B**) rectal temperatures for the animals over the course of the experiment. **C**, **D**) white cell counts on the indicated days post infection, calculated for each animal as % white cell count on day 0. E-H show examples of pathology from VU19: **E**) Inflamed coronary band on hoof. **F**) zebra striping of caecum. **G**) haemmorrhage of gums. **H**) swollen spleen.

Each virus isolate was passaged a further two times in BHK21/clone13 cells to prepare stock, and 10^4^ TCID_50_ units of virus were injected subcutaneously into 3 sheep per isolate. The animals infected with GV isolate IG619 showed higher and more prolonged pyrexia as well as profound leucopoenia (Figure 4). One of this group became extremely weak and apathetic by 7 dpi, developing clear hyperaemia in the coronary band (Figure 4), with gum lesions and bloody diarrhoea; this animal was euthanised at this point for post-mortem. A second animal with less severe clinical signs from the same group, and one animal from the group infected with the tissue culture-adapted NSDV isolate were sacrificed and post-mortem examination carried out at the same time. The animal with severe clinical signs had an enlarged spleen and multiple internal haemorrhages on blood vessels and the lining of the lower gut (Figure 4), while the other animal from the same group showed less in the way of pathology. The animals infected with NSDV-Entebbe showed only a transient pyrexia and leucopoenia, and the animal killed at 7 dpi showed no pathology at post mortem. The remaining animals were kept for a further four days, by which time temperatures had returned to normal.

**Figure 5:**
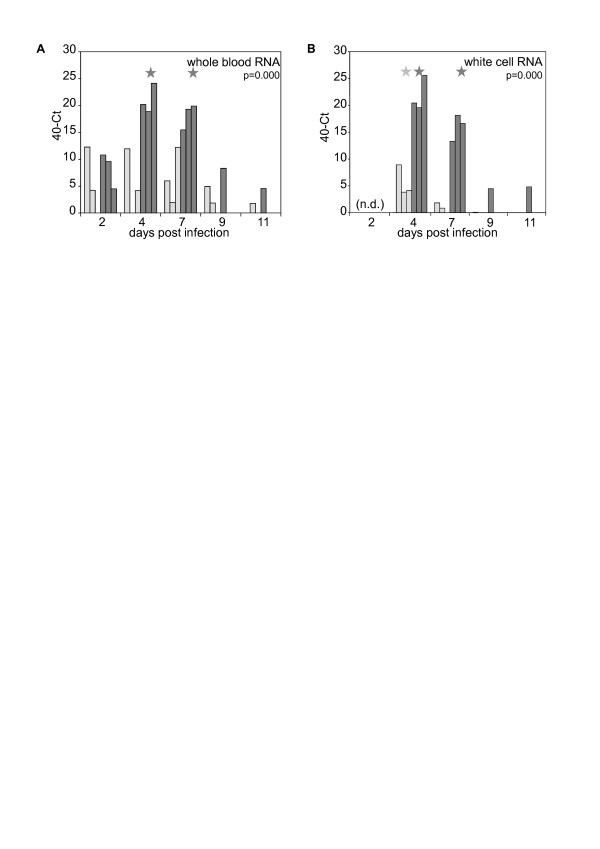
**Real-time PCR assay of viral genome RNA.** Viral genome was quantitated in (**A**) whole blood and (**B**) white cells isolated on different days post infection, for animals infected with NSDV isolate (light bars) or GV isolate (dark bars). Values are expressed as 40-Ct so that an increase in value corresponds to an increase in viral RNA. n.d. = not determined (samples lost before assay). The probability (p) value shown is that for the contrast of the two virus isolates and indicates the probability that the differences arose by chance. A star above a group of bars for a particular combination of virus and dpi indicate a significant difference from 0 at a threshold of *p* = 0.05.

RNA was prepared from whole blood samples taken directly into RNA stabilising solution (Ambion “Tempus” vacutainers) and the relative level of viral genome RNA in each sample determined. Due to the small sample numbers and the wide variation in virus load observed in different animals, not all days in which virus genome was detected appeared statistically different from zero, but the pattern of responses was nevertheless clear. Viral RNA levels were higher in the GV IG619-infected animals, peaking at around 4 dpi and falling rapidly after 7 dpi (Figure 5A). Similar results were obtained from RNA isolated from a crude white cell preparation (Figure 5B) consisting of blood from which red cells were removed (buffy coat & plasma). Virus isolation from this preparation was successful for GV at days of peak viral RNA, but the NSDV isolate could not be recovered. Several white cell samples from NSDV and GV-infected animals were found to be extremely toxic to cell cultures.

**Figure 6:**
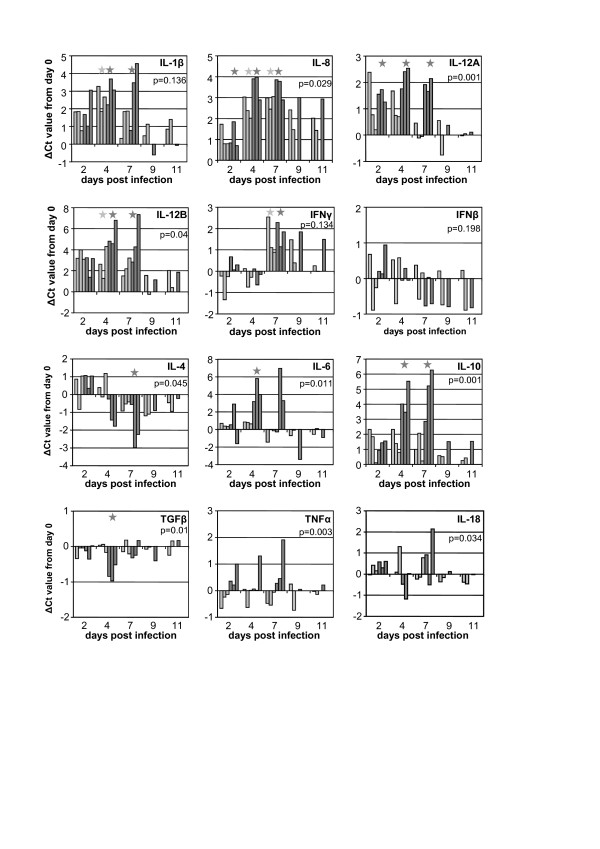
**Effects of infection on transcription of cytokine mRNAs.** Cytokine mRNA levels were determined in total blood RNA from animals infected with the NSDV isolate (light bars) or the GV isolate (dark bars). Due to the variable initial Cts seen in samples from different animals, values for day N are expressed as Ct(day 0)-Ct(day N), so that an increase in specific mRNA appears as an increase in the plotted value. The probability (*p*) value shown is that for the contrast of the two virus isolates and indicates the probability that the differences arose by chance. A star above a group of bars for a particular combination of virus and dpi indicate a significant difference from 0 at a threshold of *p* = 0.05.

The RNA prepared from whole blood which had been stabilised with complete cell lysis immediately on isolation was used to study cytokine mRNA levels during the course of infection (Figure 6). Infection with either isolate led to rapid increases in levels of IL-1β, IL-8 and IL-12 mRNA, with a later increase in IFN-γ mRNA levels as the infection was resolving. The pathogenic virus isolate caused a noticeably higher level of transcription of IL-6, IL-10 and TNFα mRNAs, and a clear if transient suppression of transcription of IL-4 and TGFβ. No consistent effect was seen in the levels of IL-18 or IFNβ mRNA in the animals of either group. A set of three housekeeping genes (glyceraldehyde phosphate dehydrogenase (GAPDH), glucose-6-phosphate dehydrogenase (G6PDH) and succinate dehydrogenase (SDHA)) showed no variation between samples (not shown), indicating that RNA recovery and the RT reactions had not introduced any significant bias into the results.

## Discussion

It is clear from the studies in tissue culture that the NSDV isolate has adapted in some way to allow it to grow well in most of the cell lines tested. At the same time, this isolate has essentially lost virulence in sheep. These findings are in accord with those of Terpstra [6], who found that NSDV of the 55^th^ to 71^st^ tissue culture passage had greatly reduced virulence, while generating a protective immune response in some animals. The nature of the attenuation remains to be determined. The attenuated virus clearly still grows in animals, though less than the pathogenic virus. This is not due to a defect in the replication machinery or assembly of the attenuated virus, as it is clear from the tissue culture studies that this virus replicates well; direct comparisons in which the two isolates are used to infect a compatible cell line (Vero cells) have shown that the NSDV isolate appeared to produce new viral protein and progeny virions slightly faster than the pathogenic isolate. Other studies in our laboratory have shown that both isolates block the actions of type 1 and type 2 interferons and the induction of type 1 interferon [12] equally well, suggesting that the decreased pathogenicity of the NSDV isolate is not associated with any loss of function in this area. One possible difference between the two isolates is a change in one or both surface glycoproteins of the virus to allow the adapted isolate to enter the cell lines tested more easily, but which has reduced the effectiveness of the virus at growing in the natural target cells in the animal. Further studies to identify the native receptor NSDV/GV are required before we can examine the receptor preference of these two isolates.

There have been no detailed studies on the nature of the pathogenesis in GV/NSDV infections; GV has only recently been identified as a widespread infection in India [3,9], and it is likely that the virus has been, in the past, frequently ignored or confused with diseases having similar signs in sheep/goats (e.g. peste des petits ruminants, Rift Valley fever), on either continent. The pyrexia seen here with the pathogenic isolate is similar to that reported previously [1,6]; the profound leucopoenia has not previously been reported for NSDV infections, although it is a common clinical sign of viral hemorraghic fever, and may be caused by the same large scale apoptosis of leukocytes seen in CCHFV-infected mice [22] or Ebola virus haemorrhagic fever [23]. Loss of white cells has been reported in CCHFV-infected humans [24].

The cytokine responses observed in this study suggest a similar pattern to that seen in CCHFV infections in humans (reviewed in [25]) and in some other haemorrhagic fevers. The pathogenesis of CCHFV is poorly understood, not least because most cases occur in areas with limited clinical pathology facilities, and work on the disease requires specialized buildings and equipment (BSL4 containment). Nevertheless, serology on CCHF patients has shown increases in IL-6 and IL-10 and increased TNFα in clinically severe (hospitalised) cases [26,27], and monocyte-derived dendritic cells infected with CCHFV release IL-6, IL-10 and TNFα [28], while we showed that pathogenic NSDV/GV was associated with increases in these cytokines as well as of IL-12, and a decrease in IL-4, all concordant with a Th1, proinflammatory response, which has been proposed for CCHFV [26,29]. One study found reduced levels of IL-12 in CCHF patients [30], but this may be a matter of timing, since the levels of IL-12 in NSDV/GV infection declined rapidly after 7 days. The observed cytokine responses would be expected to give rise to lymphohistocytosis (often associated with CCHF [29]), while both IL-6 and TNFα are associated with the increase in endothelial permeability that is common in viral hemorrhagic fevers [31,32]. Elevated TNFα is found in a number of other hemorrhagic fevers, including infection with Hantaan virus [33], Ebola virus [34] or Puumala virus [35]. It does need to be pointed out that most of those studies have measured serum cytokine proteins, while in this instance we have looked only at the levels of specific mRNAs, since specific assays for ovine cytokines have not yet been developed. This means that we will have missed some changes due to cytokines secreted by other organs (e.g. IL-6 produced by the liver); on the other hand, the real-time PCRs are very sensitive, and the serial samples allow us to pick up quite small changes in transcription patterns.

The real-time PCR detection of viral genome was much more sensitive than virus isolation, as has been seen with other viruses. Interestingly, white cell RNA was almost as sensitive as whole blood RNA for detecting virus, especially the more wild-type, pathogenic isolate, despite the fact that low yields of RNA from the white cell preparation meant that it was necessary to use less of this RNA in the RT-PCR than whole blood RNA, suggesting that the viral RNA in the blood is mostly associated with white cells, and that EDTA blood or other anticoagulated blood will be a suitable sample for laboratory testing/diagnosis.

## Competing interests

The authors declare that they have no competing financial or non-financial interests.

## Authors’ contributions

AbT processed all the samples from the animal study and carried out all the real-time PCR studies. LL prepared endothelial cells and carried out all the studies on virus growth in cell culture. BH characterised and sequenced the virus isolates. MDB conceived of, designed and directed the study, carried out the animal work and prepared the manuscript. All authors read and approved the final manuscript.
